# Isolated Superior Mesenteric Artery Dissection following Blunt Trauma: A Case Report

**DOI:** 10.1055/s-0043-1770955

**Published:** 2023-07-10

**Authors:** Katsudai Shirakabe, Masaki Kanzaki

**Affiliations:** 1 Tokyo Bay Urayasu Ichikawa. Department of General Surgery, Tokyo Bay Urayasu Ichikawa Medical Center, Urayasu-City, Chiba, Japan; 2 Department of General Surgery, Tokyo Bay Urayasu Ichikawa Medical Center, Urayasu-City, Chiba, Japan

**Keywords:** abdominal pain, blunt trauma, ischemia, ISMAD, necrosis, vomiting

## Abstract

Isolated superior mesenteric artery dissection (ISMAD) is rare, with symptoms ranging from asymptomatic to acute intestinal ischemia. Risk factors for ISMAD include hypertension, atherosclerosis, abnormal elastic fibers, and pregnancy. In the present case, blunt trauma, which has not been previously reported, was suggested as a risk factor. A 46-year-old man was brought to the emergency room after he was found unconscious after a motor vehicle collision. At presentation, he had no abdominal symptoms; however, on the fourth day of admission, he presented with severe abdominal pain and vomiting. A contrast-enhanced computed tomography scan revealed an ISMAD with associated intestinal ischemia and necrosis; hence, emergency surgery was performed. Herein, we report a case of ISMAD caused by blunt abdominal trauma.

Arterial dissection involves the destruction of the arterial wall. This begins with a tear in the tunica intima, which progresses to bleeding in the tunica media, forming a false lumen adjacent to the true lumen. The dissected flap blocks the opening of the branching artery causing ischemia of the distal organs. Furthermore, blood in the false lumen can coagulate and cause thromboembolism.

Isolated superior mesenteric artery dissection (ISMAD) without aortic dissection has been reported as a symptomatic or incidental finding since 1947. 1

ISMAD is particularly rare, with a reported incidence of 0.06% in 66,666 autopsy cases. 2 Progress in imaging techniques has led to an increasing number of cases of spontaneous ISMAD being detected.

Vascular disease and anatomical problems have been considered the most common causes of dissection of the superior mesenteric artery (SMA); however, trauma is extremely rare. Herein, we report such a rare case of ISMAD caused by blunt abdominal trauma.

## Case Presentation

A 46-year-old man was brought to the emergency room after an emergency medical team found him unconscious in a car. He had lost consciousness while driving on a highway and hit a guardrail. The patient had a history of epilepsy.

Upon arrival at the hospital, his consciousness was clear, and his vital signs were as follows: blood pressure 150/87 mm Hg, pulse 88/min, respiratory rate 18/min, and body temperature 36.3°C. A computed tomography (CT) scan revealed fractures of the L1 vertebral body, transverse process, and right 11th rib; hence, the patient was hospitalized for treatment. A contrast CT scan was not performed; thus, SMA dissection was unknown at this time.

**Fig. 1:**
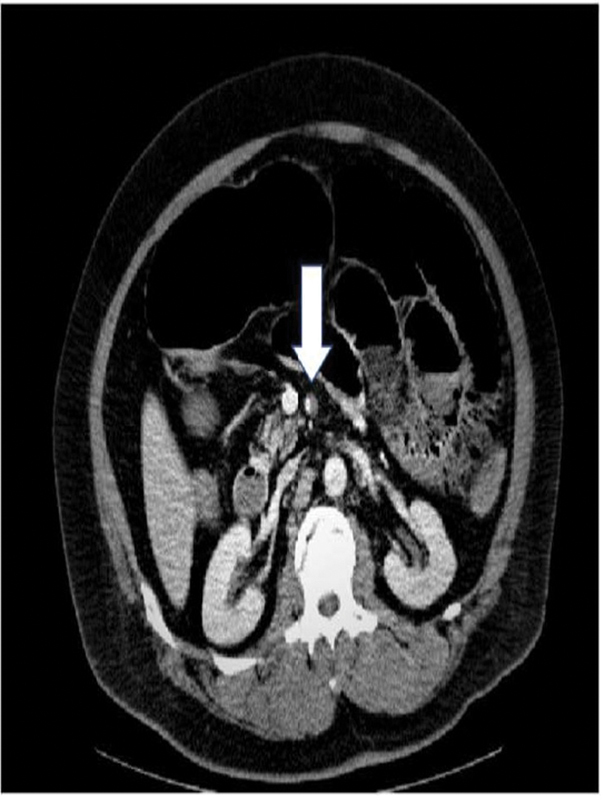
White arrow indicate dissociation of the superior mesenteric artery. Pneumatosis intestinalis is observed (the white arrow indicates a dissected SMA).

**Fig. 2:**
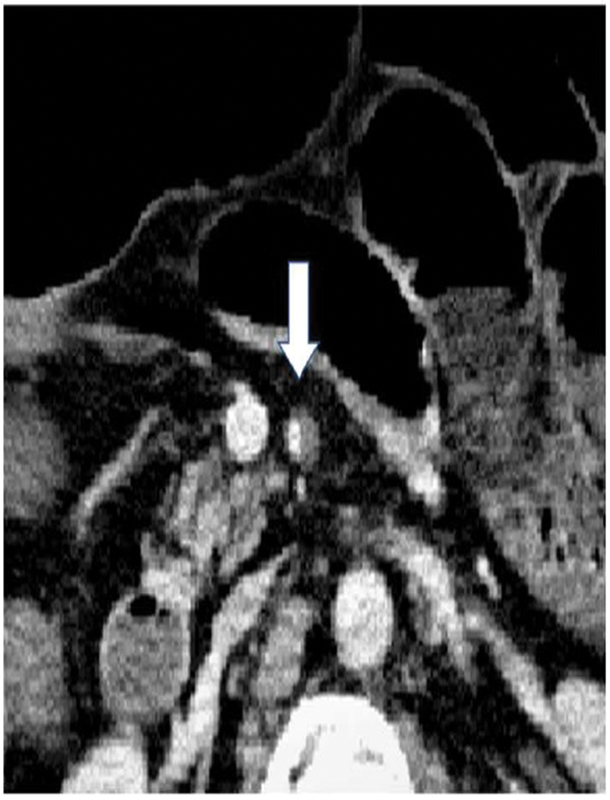
Dissection of the superior mesenteric artery is observed, with hematoma occlusion of the false lumen.

**Fig. 3:**
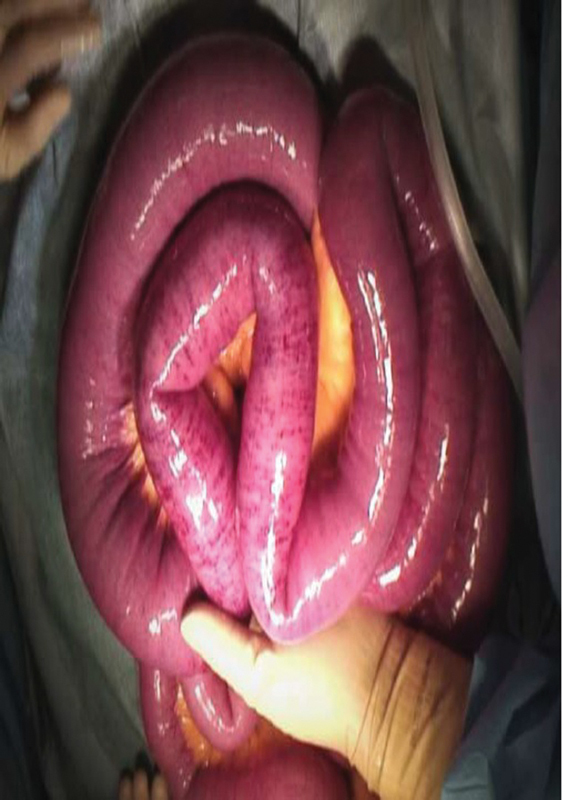
The small bowel shows patchy ischemia in a 25-cm area 100 cm from the Treitz ligament.

Following admission, abdominal distention and constipation were observed, and the patient was treated with laxatives. On the fourth day of hospitalization, the patient experienced abdominal pain and vomiting after breakfast. A contrast-enhanced CT scan revealed intramural pneumatosis of the small bowel, portal vein pneumatosis, and dissection of the SMA ( Fig. 1 ). The patient was in a state of panperitonitis, and emergency surgery was suggested for ischemia and necrosis of the small intestine due to dissection of the SMA ( Fig. 2 ). The patient was sent to the operation room, and during the surgery, the small intestine was found to be poorly colored for 25 cm starting from a point 100 cm from the ligament of Treitz ( Fig. 3 ); therefore, partial resection of the small bowel was performed.

A second-look surgery was performed 2 days after the first surgery, and because there was no progression of necrosis, anastomosis of the small bowel was performed. Abdominal symptoms did not recur after surgery.

## Discussion

ISMAD was first reported in 1947. 1 An increasing number of patients with ISMAD have been reported in recent years because of the increasing use of contrast-enhanced CT scans.

The etiology of ISMAD is associated with fibromuscular dysplasia, medial degeneration, and atherosclerosis and is rarely due to trauma. 3

Injuries to the SMA, which generally account for less than 1% of cases in trauma centers, are rare and fatal, with most injuries being detected by hematoma formation. 4

The structure of the SMA is another etiologic factor, and certain deformations increase its susceptibility to shear stress, consequently making it more prone to detachment. Shear stress occurs when the SMA loses its mechanical support from the pancreas and bends freely within the mesenteric root. 5 Furthermore, it has been observed that as the angle between the aorta and SMA approaches 90 degrees, the shear stress, and the incidence of ISMAD increase. Hemodynamics are accelerated at the SMA transition, causing abnormal mechanical stress on the anterior wall, and inducing dissection. 6 7

There are four main classification systems of ISMAD, all of which are based on imaging findings. The oldest classification is that of Sakamoto et al, 8 which divides ISMAD into four types, types I to IV. Zerbib et al 9 modified Sakamoto et al's classification, adding type V with aneurysmal dissection and distal SMA stenosis and types VIa and VIb with total and partial SMA thrombi. A more convenient classification is Yun et al's classification, 10 which classifies ISMAD into three categories based on morphology. Another classification is Luan and Li's classification, 11 which classifies ISMAD based on site rather than morphology.

All these classifications are based on the imaging appearance of ISMAD and whether the true or false lumen is occluded or thrombosed.

The classification of this disease was type II according to Yun et al. Although the type of the disease was relatively unlikely to cause ischemia, ischemia and necrosis due to disruption of blood flow occurred.

Further studies are needed to use as a guide for ISMAD treatment and the need for intervention now depends on the clinical presentation.

The clinical presentation of ISMAD ranges from asymptomatic to acute peritonitis. The most common symptom is abdominal pain. Other atypical accompanying symptoms include nausea and vomiting, diarrhea, abdominal distention, backache, bloody stool, anorexia, constipation, weight loss, fever, hyperhidrosis, retrosternal pain, and chest tightness. 12 The goals of ISMAD treatment are to relieve symptoms and prevent intestinal necrosis and SMA rupture. Conservative treatment is the most common initial treatment modality, performed in 68.0 13 to 71.8% 14 of patients. Symptom relief rates range from 74.6 13 to 86.6%. 14 Generally, if bowel necrosis, rupture, or aneurysmal SMA is not present, conservative treatment is safe and effective. However, some patients with ISMAD may develop intestinal ischemia secondary to thrombus formation and subsequent thrombus occlusion. Therefore, some studies recommend administering antiplatelet and anticoagulant medications in the medical treatment of ISMAD. 15 Endovascular repair procedures, such as stenting and false lumen coiling, exist as treatment options for ISMAD, but the indications remain unclear. Failure of conservative treatment, which accounts for 16% of cases, is the most common reason for conversion to endovascular repair. 16 In the presence of peritonitis, endovascular stenting with laparoscopy or laparotomy, or a combination of both, may be a reasonable option.

ISMAD due to blunt trauma is rare, and no definitive treatment exists. Although there are many reports of successful conservative treatment, in this case, there were signs of ischemia and necrosis of the small bowel, and emergency surgery was required. It is important to determine the need for intervention as soon as possible after diagnosis.

## Conclusion

In this case, the lack of a contrast-enhanced CT scan at the time of injury led to a delay in diagnosis. Therefore, performing diagnostic workup early after a major injury is important to rule out solid organ injuries. The incidence of ISMAD is increasing with the advancement and availability of imaging technology. Various classifications of ISMAD exist based on imaging findings, but there remains no classification that directly relates to clinical treatment. Although certain vascular diseases and anatomical problems are possible causes of ISMAD, trauma is rare. ISMAD is generally relieved with conservative treatment, a small number of patients require endovascular treatment and surgery and should be carefully monitored.
